# Familial Hypercholesterolemia: Real-World Data of 1236 Patients Attending a Czech Lipid Clinic. A Retrospective Analysis of Experience in More than 50 years. Part II. Clinical Characteristics

**DOI:** 10.3389/fgene.2022.849267

**Published:** 2022-03-14

**Authors:** Tereza Altschmiedova, Veronika Todorovova, Michal Vrablik, Richard Ceska

**Affiliations:** Third Department of Medicine - Department of Endocrinology and Metabolism of the First Faculty of Medicine, Charles University and General University Hospital, Prague, Czechia

**Keywords:** familial hypercholesterolemia, LDL-cholesterol, Lp(a), ASCVD, RWD

## Abstract

**Introduction:** Patients with familial hypercholesterolemia (FH) are at increased risk of premature atherosclerotic cardiovascular disease (ASCVD).

**Aim of study:** To perform a retrospective analysis of data to assess the effects of individual lipoproteins and other risk factors (RFs) on the development of ASCVD and to compare these parameters in individuals with versus without ASCVD.

**Patients and methods:** Our study group included a total of 1,236 patients with FH (395 men and 841 women with a mean age of 44.8 ± 16.7 years) attending a single lipid clinic. The diagnosis of FH was established using the Dutch Lipid Clinic Network score (DLCN). Among the 1236 FH patients, 1,008 of them [854 suspected with LDL receptor-mediated FH and 154 with familial defective apolipoprotein B-100 (FDB)] were genetically analysed. Their RFs were assessed based on the patients’ clinical characteristics.

**Results:** While patients with ASCVD had higher baseline LDL-C, TC, TG and Lp(a) compared with patients without this diagnosis, this ratio was just the opposite by the follow-up. The highest statistically significant differences were seen in the baseline levels of Lp(a) and, quite surprisingly, TG. Except for Lp(a), the levels of all lipid parameters declined significantly over time. While the incidence of diabetes and arterial hypertension was not higher in our group compared with the general population, these patients were at a more significant risk of ASCVD.

**Conclusion:** Familial hypercholesterolemia is a major RF for the development of ASCVD. While our analysis confirmed the important role of LDL-C, it also corroborated a strong correlation between ASCVD and other lipid parameters, and Lp(a) and TG in particular. Familial hypercholesterolemia is not the only RF and, to reduce cardiovascular risk of their patients, physicians have to search for other potential RFs. Patients diagnosed to have FH benefit from attending a specialized lipid clinic perse.

## Introduction

With an estimated prevalence of 1 to 200–250, familial hypercholesterolemia (FH) ranks among the most frequent inherited metabolic diseases (Nordestgaard et al., 2013; Beheshti et al., 2020). The typical FH patient is predestined to have high LDL cholesterol (LDL-C) levels since childhood considerably raising the risk of premature atherosclerotic cardiovascular disease (ASCVD) (The Lipid Research Clinic, 1984; Watts et al., 2016; Ference et al., 2017). All patients diagnosed with FH are automatically at least at high risk of developing ASCVD (Visseren et al., 2021). However, we suppose there are differences between individual patients which will decide whether or not ASCVD will eventually develop. All FH patients require, in particular, an early diagnosis and initiation of lipid-lowering therapy as soon as possible. The class of drugs of choice are statins which, by effectively lowering LDL-C levels, significantly reduce cardiovascular morbidity and mortality (Versmissen et al., 2008). To achieve the target levels of LDL-C, combination lipid-lowering therapy is quite often necessary; most often a combination of a statin with ezetimibe or, alternatively, with a PCSK9 inhibitor, is used (Visseren et al., 2021).

## Aim of Study

One of the goals of our project was to present the baseline and follow-up clinical and biochemical findings in a large cohort of patients diagnosed to have FH and attending a single lipid center to show that patients do benefit from mere surveillance and highly specialized therapy. In addition to assessing the effects of therapy on pre-defined lipid parameters, we evaluated the effects of individual lipoproteins and other major risk factors on the development of complications associated with the atherosclerotic process. In particular, we focused our attention on differences between the parameters in patients whose FH is already complicated by overt ASCVD and those without ASCVD in order to identify factors contributing to a complicated course of the disease.

## Patient Characteristics and Methods

The submitted project is a retrospective analysis of data of a total of 1,236 patients (841 women and 395 men with a mean age of 44.8 ± 16.7 years) with FH on follow-up in a single lipid center. The period of data collection started in the 1960s and last data were analysed in the 2020. The average follow-up time was not analysed.

Data of a large cohort of patients were analysed using multiple parameters. This article (Part II) focuses on FH clinical symptomatology. The principles of biochemical, statistical and genetic analyses of blood samples and classification of FH patients by the type of gene mutation are addressed in a Part I co-published by Todorovova et al. (2022) hence, they are not discussed more in detail in this article.

The diagnosis of FH was established using the Dutch Lipid Clinic Network score (DLCN). Among the 1,236 FH patients, 1,008 of them [854 supposed to have mutation in *LDLR* gene and 154 with familial defective apolipoprotein B-100 (FDB)] were genetically analysed (Todorovova et al., 2022).

The parameters of lipid and lipoprotein metabolism investigated in our analysis included LDL-cholesterol (LDL-C), total cholesterol (TC), apolipoprotein B (ApoB), HDL-cholesterol (HDL-C), triglycerides (TG) and apolipoprotein Lp(a). Their levels were recorded and analysed in patients at baseline in our clinic and compared with their current or latest available data. Also assessed was the presence of the other major risk factors for atherosclerosis, i.e., arterial hypertension, diabetes mellitus and smoking.

Our group of patients was further subdivided, into subgroups to be compared using several characteristics.

**FIGURE 1:**
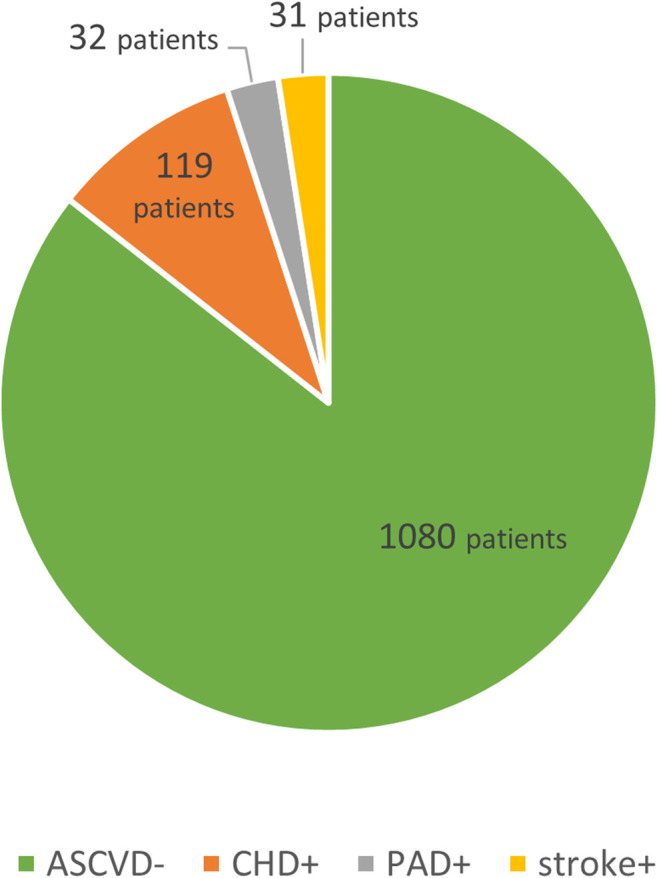
ASCVD subgroups.

The first division was based on the presence/absence of ASCVD in their history, with patients showing overt complications of the atherosclerotic process further subdivided into three subgroups by the anatomical site involved, i.e., those with coronary heart disease (CHD), ischemic cerebrovascular event (stroke) and peripheral arterial disease (PAD). See Figure 1.

**FIGURE 2:**
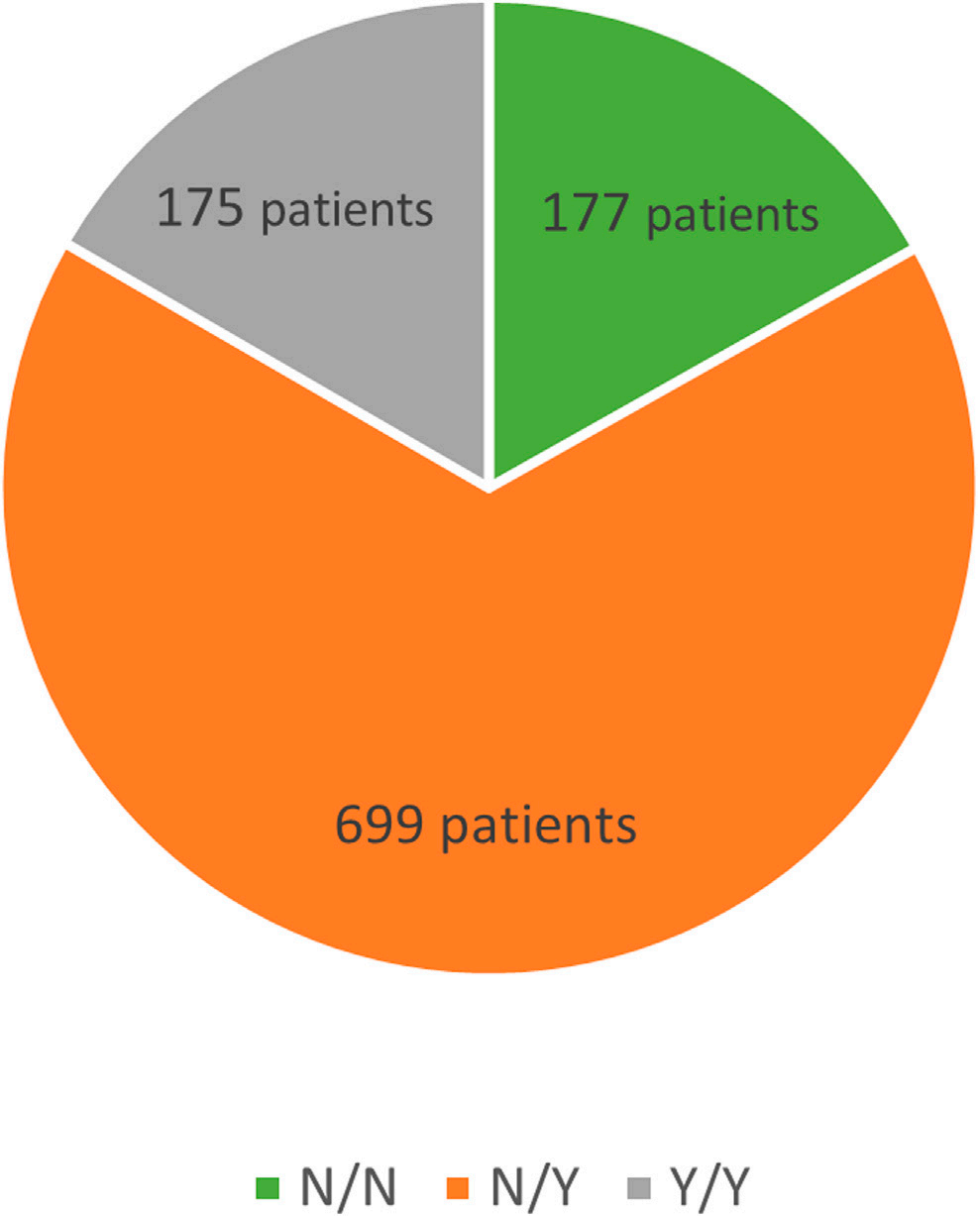
Treatment subgroups.

Another division, again into three subgroups, was based on differences in drug therapy. The first subgroup was made up of patients not taking any medications both prior to and during follow-up in our clinic, the second subgroup consisted of patients with pharmacotherapy not initiated until the start of follow-up whereas patients in the third subgroup had been on drug therapy already at baseline and continued their pharmacotherapy thereafter. See Figure 2.

Data were analyzed using STATISTICA 13 software (TIBCO Software Inc., Palo Alto, CA, United States). The baseline and follow-up levels were compared using the paired *t*-test. In univariate analysis, correlations between the lipid parameters and age were determined using Pearson’s correlation coefficients. The tests used when comparing two and three subgroups in univariate analysis were the two-sample *t*-test and ANOVA test, respectively. We used multivariable logistic regression model to assess the effect of risk factors smoking, diabetes and arterial hypertension for total cardiovascular risk.

## Results

The present analysis compared the levels of lipid parameters obtained prior to start of follow-up and the most recent ones available. The primary endpoint LDL-C declined from a baseline mean of 6.49 ± 1.92 mmol/L to 3.26 ± 1.57 mmol/L (by 49.8%). A decrease by 39% was observed in TC levels falling from 8.95 ± 1.95 mmol/L to 5.43 ± 1.69 mmol/L. ApoB showed a decrease from a baseline mean of 1.76 ± 0.56 mmol/L to 1.09 ± 0.56 mmol/L. TG levels declined from a mean baseline of 1.81 ± 1.13 mmol/L to 1.38 ± 0.78 mmol/L. The change in HDL-C levels was 1.67 ± 0.46 mmol/L vs. follow-up levels of 1.56 ± 0.46 mmol/L. All the above differences were significant (*p* < 0.001). Lp(a) was unchanged (0.56 vs. 0.59 g/L, *p* = 0.27).

A total of 156 patients of the entire group (12.6%) had a history of ASCVD (ASCVD+ group; mean age 54.0 ± 12.5; 89 women, 67 men; 75 smokers) in the form of either CHD, stroke or PAD. As a total of 1,080 patients were in primary prevention of ASCVD, atherosclerosis had not yet manifested itself (ASCVD–group; mean age 43.5 ± 16.8; 752 women, 328 men; 313 smokers). The primary outcome was LDL-C declining, in ASCVD+ (ASCVD–) patients, from a baseline 6.85 ± 2.05 (6.42 ± 1.89) mmol/L to 2.79 ± 1.54 (3.23 ± 1.57) mmol/L during follow-up, which was 60% (48%) difference. This trend was seen in TC levels either, which fell in the ASCVD+ (ASCVD–) subgroups by 48% (38%). While the differences between the two subgroups in the baseline levels of ApoB were non-significant, follow-up difference reached statistical significance. The baseline TG levels of patients with a history of ASCVD were higher compared with patients without ASCVD. The TG levels decreased in either subgroup, 34% in ASCVD+, and 21% in ASCVD–patients. Statistically significant were the differences in baseline Lp(a) levels. In ASCVD+ subgroup, Lp(a) levels decreased, whereas in ASCVD-subgroup increased towards follow-up. HDL-C levels decreased over time, the overall change from baseline to follow-up was non-significant. For more details see Table 1.

**TABLE 1:** Patients with/without ASCVD and effect of treatment on lipid levels.

Parameter	ASCVD	Baseline			Follow-up			N	Diference (%)	*p*
		N	Mean ± SD	*p*	N	Mean ± SD	*p*			
**LDL-C (mmol/L)**	+	146	6.85 ± 2.05	*p* = 0.011	149	2.79 ± 1.54	*p* < 0.001	140	−59.72	*p* < 0.001
	-	1,035	6.42 ± 1.89		937	3.32 ± 1.57		909	−48.09	
**TC (mmol/L)**	+	156	9.28 ± 2.13	*p* = 0.012	153	4.83 ± 1.69	*p* < 0.001	153	−47.95	*p* < 0.001
	-	1,071	8.86 ± 1.93		965	5.53 ± 1.67		965	−37.84	
**ApoB (g/L)**	+	77	1.87 ± 0.62	*p* = 0.137	48	0.96 ± 0.43	*p* = 0.05	20	−48.03	*p* = 0.219
	-	525	1.77 ± 0.53		314	1.11 ± 0.50		164	−36.70	
**TG (mmol/L)**	+	153	2.11 ± 1.36	*p* < 0.001	153	1.39 ± 0.73	*p* = 0.905	150	−34.26	*p* < 0.001
	-	1,064	1.74 ± 1.06		964	1.38 ± 0.79		958	−21.48	
**HDL-C (mmol/L)**	+	153	1.55 ± 0.42	*p* = 0.001	152	1.43 ± 0.45	*p* < 0.001	149	−8.36	*p* = 0.578
	-	1,057	1.68 ± 0.46		955	1.58 ± 0.46		943	−6.57	
**Lp(a) (g/L)**	+	108	0.66 ± 0.79	*p* < 0.001	36	0.77 ± 1.08	*p* = 0.107	36	−8.35	*p* = 0.123
	-	844	0.44 ± 0.58		253	0.55 ± 0.67		248	7.62	

N—number of patients; SD, standard deviation; *p*—*p*-value.

Patients with ASCVD (ASCVD+; *n* = 156) were further subdivided into three subgroups by the anatomical site involved into those with CHD (*n* = 119), stroke (*n* = 31) and PAD (*n* = 32). Some patients were included in more than one subgroup. Figure 1.

All results are summarized in Table 2. In the CHD+ subgroup, LDL-C levels decreased by 60% from a baseline during follow-up compared with CHD–patients without a history of CHD (CHD–), whose baseline fell by 49%. In the CHD+ (CHD–) subgroups, TC levels decreased by 48% (38%). The differences in the levels of ApoB between the individual subgroups were non-significant both at the start and during follow-up. The baseline TG levels in the CHD+ (CHD–) subgroups were 2.12 ± 1.47 (1.75 ± 1.05) mmol/L to be non-significant during follow-up. Patients in the CHD+ subgroup had lower baseline levels of HDL-C compared with CHD–patients. Lp(a) levels were higher at baseline in CHD+ patients compared with CHD-. These levels rose in both subgroups over time, the changes were not significant (*p* = 0.801).

**TABLE 2:** Patients with/without CHD/stroke/PAD and effect of treatment on lipid levels.

Parametr	Group	Baseline			Follow-up			N	Diference (%)	*p*
		N	Mean ± SD	*p*	N	Mean ± SD	*p*			
**LDL-C (mmol/L)**	CHD+	110	6.90 ± 2.21	*p* = 0.015	113	2.82 ± 1.57	*p* = 0.002	105	−59.75	*p* < 0.001
	CHD-	1,071	6.43 ± 1.88		973	3.30 ± 1.57		944	−48.53	
	stroke+	31	6.84 ± 1.65	*p* = 0.282	30	2.57 ± 1.20	*p* = 0.016	30	−62.29	*p* = 0.007
	stroke-	1,150	6.47 ± 1.93		1,056	3.27 ± 1.58		1,019	−49.33	
	PAD+	31	7.14 ± 1.86	*p* = 0.051	32	2.99 ± 1.61	*p* = 0.342	31	−57.61	*p* = 0.017
	PAD-	1,150	6.46 ± 1.92		1,054	3.26 ± 1.58		1,018	−49.46	
**TC (mmol/L)**	CHD+	119	9.31 ± 2.29	*p* = 0.020	116	4.88 ± 1.73	*p* < 0.001	116	−47.57	*p* < 0.001
	CHD-	1,108	8.87 ± 1.92		1,002	5.50 ± 1.67		1,002	−38.27	
	stroke+	31	9.46 ± 1.78	*p* = 0.115	31	4.60 ± 1.33	*p* = 0.005	31	−51.40	*p* < 0.001
	stroke-	1,196	8.90 ± 1.97		1,087	5.46 ± 1.69		1,087	−38.91	
	PAD+	32	9.54 ± 1.97	*p* = 0.069	32	4.94 ± 1.75	*p* = 0.096	32	−48.16	*p* = 0.006
	PAD-	1,195	8.90 ± 1.96		1,086	5.45 ± 1.68		1,086	−39.00	
**ApoB (g/L)**	CHD+	57	1.88 ± 0.66	*p* = 0.135	34	0.98 ± 0.44	*p* = 0.149	15	−43.20	*p* = 0.741
	CHD-	545	1.77 ± 0.53		328	1.11 ± 0.50		169	−37.46	
	stroke+	16	1.78 ± 0.40	*p* = 0.968	11	0.79 ± 0.36	*p* = 0.039	4	−68.20	*p* = 0.033
	stroke-	586	1.78 ± 0.54		351	1.10 ± 0.50		180	−37.17	
	PAD+	17	2.01 ± 0.57	*p* = 0.081	9	1.10 ± 0.37	*p* = 0.954	4	−51.01	*p* = 0.242
	PAD-	585	1.77 ± 0.54		353	1.09 ± 0.50		180	−37.57	
**TG (mmol/L**)	CHD+	118	2.12 ± 1.47	*p* < 0.001	116	1.42 ± 0.78	*p* = 0.623	115	−33.55	*p* = 0.002
	CHD-	1,099	1.75 ± 1.05		1,001	1.38 ± 0.78		993	−22.10	
	stroke+	30	2.02 ± 0.85	*p* = 0.235	31	1.29 ± 0.46	*p* = 0.523	30	−35.74	*p* = 0.119
	stroke-	1,187	1.78 ± 1.11		1,086	1.39 ± 0.79		1,078	−23.13	
	PAD+	31	2.06 ± 0.83	*p* = 0.155	32	1.46 ± 0.67	*p* = 0.562	31	−28.87	*p* = 0.365
	PAD-	1,186	1.78 ± 1.11		1,085	1.38 ± 0.78		1,077	−23.33	
**HDL-C (mmol/L)**	CHD+	118	1.54 ± 0.43	*p* = 0.002	116	1.42 ± 0.45	*p* < 0.001	115	−7.87	*p* = 0.825
	CHD-	1,092	1.68 ± 0.46		991	1.57 ± 0.46		977	−6.68	
	stroke+	30	1.58 ± 0.44	*p* = 0.304	30	1.48 ± 0.52	*p* = 0.355	29	−7.39	*p* = 0.947
	stroke-	1,180	1.67 ± 0.46		1,077	1.56 ± 0.46		1,063	−6.78	
	PAD+	31	1.57 ± 0.41	*p* = 0.236	32	1.29 ± 0.34	*p* = 0.001	31	−17.22	*p* = 0.023
	PAD-	1,179	1.67 ± 0.46		1,075	1.56 ± 0.46		1,061	−6.51	
**Lp(a) (g/L)**	CHD+	80	0.72 ± 0.81	*p* < 0.001	25	0.96 ± 1.24	*p* = 0.007	25	0.71	*p* = 0.801
	CHD-	872	0.44 ± 0.58		264	0.54 ± 0.66		259	5.31	
	stroke+	17	0.53 ± 0.84	*p* = 0.634	6	0.46 ± 0.36	*p* = 0.677	6	−53.56	*p* < 0.001
	stroke-	935	0.46 ± 0.60		283	0.58 ± 0.74		278	6.85	
	PAD+	23	0.66 ± 0.65	*p* = 0.112	9	0.43 ± 0.44	*p* = 0.521	9	2.96	*p* = 0.916
	PAD-	929	0.46 ± 0.61		280	0.59 ± 0.74		275	4.67	

N—number of patients; SD, standard deviation; *p*—*p*-value.

In patients with a history of stroke (stroke+), no significant differences in the baseline levels were found. The follow-up LDL-C (as well as TC or ApoB) levels in patients stroke+ were lower than in subgroup without this condition (stroke-).

While patients with PAD did not show significant differences in the lipid parameters at baseline, a significant difference was noted over time in HDL-C levels, being lower in PAD+ patients compared with PAD–subgroup.

Among the 1,236 patients, drug-status was available for 1,051 patients, and these were then subdivided into three subgroups based on whether or not the patients had been previously on lipid-lowering therapy and whether or not they were currently being treated with lipid-lowering agents.

The Y/Y subgroup (*n = 175*, lipid-lowering therapy at baseline and during follow-up) had baseline LDL-C levels of 5.76 ± 1.93 mmol/L decreasing to 2.89 ± 1.13 mmol/L (a 50% reduction; *p* < 0.001). The baseline TC levels of 8.15 ± 1.98 mmol/L declined to 5.03 ± 1.31 mmol/L (by 38%; *p* < 0.001) during follow-up, TG levels decreasing by 23% (*p* = 0.026).

The N/Y subgroup (*n = 699,* no therapy at baseline, therapy during follow-up) showed a decrease in LDL-C from baseline of 6.83 ± 1.80 mmol/L to 3.01 ± 1.37 mmol/L (a reduction by 56%; *p* < 0.001). TC levels dropped from 9.32 ± 1.83 mmol/L to 5.16 ± 1.49 mmol/L (down by 45%; *p* < 0.001). The levels of TG declined during follow-up by 26% (*p* = 0.026).

In the N/N subgroup (*n = 177*, therapy-naïve at baseline and no therapy during follow-up), the baseline LDL-C levels of 6.35 ± 2.07 mmol/L declined to 5.48 ± 2.02 mmol/L (11% reduction; *p* < 0.001), TC levels decreased from 8.72 ± 2.07 mmol/L to 7.84 ± 1.90 mmol/L (by 9%; *p* < 0.001) and TG levels reduction was 12% (*p* = 0.026).

More details in Table 3. When comparing the values between the three subgroups, the biggest decrease (*p* < 0.001) occurred in the LDL-C, TC, ApoB, HDL-C and TG levels in the N/Y subgroup (*p* = 0.026). The differences in Lp(a) levels were non-significant. The smallest changes were documented among N/N patients showing significantly (*p* = 0.003) lowest baseline TG levels compared with the Y/Y and N/Y subgroups. The follow-up levels of LDL-C, TC and ApoB were highest in the N/N subgroup (*p* < 0.001). All results summarized in a table are available in Todorovova et al., 2022

**TABLE 3:** Distribution of FH patients by treatment and effect of treatment on lipid levels.

Parameter	Group	Baseline			End of study			N	Diference (%)	*p*
		N	Mean ± SD	*p*	N	Mean ± SD	*p*			
**LDL-C (mmol/L)**	Y/Y	167	5.76 ± 1.93	*p* < 0.001	166	2.89 ± 1.13	*p* < 0.001	160	−49.6	*p* < 0.001
	N/Y	678	6.83 ± 1.80		678	3.01 ± 1.37		660	−55.7	
	N/N	172	6.35 ± 2.07		97	5.48 ± 2.02		93	−10.7	
**TC (mmol/L)**	Y/Y	175	8.15 ± 1.98	*p* < 0.001	169	5.03 ± 1.31	*p* < 0.001	169	−38.2	*p* < 0.001
	N/Y	699	9.32 ± 1.83		689	5.16 ± 1.49		689	−44.8	
	N/N	177	8.72 ± 2.07		102	7.84 ± 1.90		102	−8.8	
**APOB (g/L)**	Y/Y	97	1.57 ± 0.51	*p* < 0.001	65	0.98 ± 0.34	*p* < 0.001	43	−37.0	*p* < 0.001
	N/Y	316	1.86 ± 0.51		190	0.99 ± 0.39		86	−45.4	
	N/N	89	1.85 ± 0.65		40	1.69 ± 0.79		14	−7.9	
**TG (mmol/L)**	Y/Y	174	1.86 ± 1.17	*p* = 0.003	169	1.41 ± 0.69	*p* = 0.792	168	−23.4	*p* = 0.026
	N/Y	690	1.85 ± 1.17		688	1.37 ± 0.80		680	−26.4	
	N/N	177	1.54 ± 0.84		102	1.38 ± 0.84		102	−11.8	
**HDL-C (mmol/L)**	Y/Y	174	1.63 ± 0.39	*p* = 0.536	169	1.51 ± 0.40	*p* < 0.001	168	−6.9	*p* < 0.001
	N/Y	687	1.67 ± 0.45		682	1.53 ± 0.44		671	−8.2	
	N/N	175	1.67 ± 0.50		102	1.77 ± 0.54		101	1.9	
**Lp(a) (g/L)**	Y/Y	154	0.61 ± 0.66	*p* = 0.003	47	0.74 ± 0.67	*p* = 0.229	47	7.7	*p* = 0.921
	N/Y	553	0.44 ± 0.60		181	0.57 ± 0.80		180	4.8	
	N/N	113	0.39 ± 0.62		14	0.38 ± 0.39		13	12.1	

Y/Y—on treatment at baseline and throughout the study; N/Y—no treatment at baseline/on treatment throughout the study; N/N—no treatment at baseline and throughout the study; N—number of patients; SD, standard deviation; *p*—*p*-value.

Our cohort comprised of 332 patients (27%) with arterial hypertension (AH). In the subgroup of patients with this diagnosis (AH+), the baseline levels of lipid parameters were significantly higher than in the subgroup without AH (AH–) such as in TC, TG, Lp(a) and lower in HDL-C. In the AH+ subgroup, the follow-up levels were significantly lower compared with the AH–subgroup in LDL-C (2.75 ± 1.21 vs 3.46 ± 1.66 mmol/L; *p* < 0.001), TC and ApoB, whereas TG levels in the AH+ subgroup showed poorer control (1.52 ± 0.77 mmol/L) than in AH–patients (1.32 ± 0.78 mmol/L). For more details see Table 4.

**TABLE 4:** Patients with/without arterial hypertension and effect of treatment on lipid levels.

Parameter	AH	Baseline			Follow-up			N	Diference (%)	*p*
		N	Mean ± SD	*p*	N	Mean ± SD	*p*			
**LDL-C (mmol/L)**	+	319	6.64 ± 1.90	*p* = 0.073	322	2.75 ± 1.21	*p* < 0.001	310	−58.17	*p* < 0.001
	-	862	6.41 ± 1.92		764	3.46 ± 1.66		739	−46.06	
**TC (mmol/L)**	+	332	9.12 ± 1.96	*p* = 0.026	328	4.89 ± 1.35	*p* < 0.001	328	−46.33	*p* < 0.001
	-	895	8.84 ± 1.96		790	5.66 ± 1.76		790	−36.27	
**ApoB (g/L)**	+	159	1.81 ± 0.57	*p* = 0.455	90	0.96 ± 0.37	*p* = 0.003	48	−46.83	*p* = 0.122
	-	443	1.77 ± 0.53		272	1.14 ± 0.52		136	−35.48	
**TG (mmol/L)**	+	331	2.09 ± 1.14	*p* < 0.001	328	1.52 ± 0.77	*p* < 0.001	327	−27.38	*p* = 0.002
	-	886	1.67 ± 1.07		789	1.32 ± 0.78		781	−21.49	
**HDL-C (mmol/L)**	+	327	1.61 ± 0.42	*p* = 0.013	327	1.46 ± 0.42	*p* < 0.001	322	−9.37	*p* = 0.032
	-	883	1.68 ± 0.47		780	1.59 ± 0.47		770	−5.74	
**Lp(a) (g/L)**	+	250	0.58 ± 0.75	*p* < 0.001	84	0.69 ± 0.92	*p* = 0.112	84	−5.95	*p* = 0.056
	-	702	0.42 ± 0.54		205	0.54 ± 0.64		200	11.20	

AH, arterial hypertension; N—number of patients; SD, standard deviation; *p*—*p*-value.

The number of AH+ patients developing ASCVD was significantly higher (27.4%) than of those without it (AH–) (7.2%). For details see Table 5. In group AH+ is 2.44 greater chance for KVO (OR = 2.44; CI0.95 = (1.65; 3.63)) than in AH-.

**TABLE 5:** AH+/AH- patients developing ASCVD.

	AH	ASCVD +	ASCVD -	Total
**Count**	+	91	241	332
**Row Percent (%)**		27.41	72.59	
**Count**	-	65	839	904
**Row Percent (%)**		7.19	92.81	

During follow-up, diabetes mellitus was diagnosed in a total of 83 patients (7% of the whole study group; *n* = 1,236). Patients with diabetes mellitus (DM+) showed worse control of lipid parameters than those without this diagnosis (DM–) as reflected in the levels of TG and HDL-C. The differences in the other parameters assessed were non-significant. Follow-up levels of LDL-C and TC in DM+ patients were lower compared with DM–patients. On the other hand, the follow-up levels of TG were higher in the DM+ subgroup than among DM–patients. The difference in ApoB levels was not significant. All pertinent data are shown in detail in Table 6.

**TABLE 6:** Patients with/without diabetes and effect of treatment on lipid levels.

Parameter	DM	Baseline			Follow-up			N	Diference (%)	*p*
		N	Mean ± SD	*p*	N	Mean ± SD	*p*			
**LDL-C (mmol/L)**	+	77	6.52 ± 1.78	*p* = 0.827	78	2.62 ± 1.35	*p* < 0.001	73	−58.95	*p* = 0.008
	-	1,104	6.47 ± 1.93		1,008	3.30 ± 1.58		976	−49.03	
**TC (mmol/L)**	+	83	9.07 ± 1.77	*p* = 0.467	82	4.74 ± 1.49	*p* < 0.001	82	−47.63	*p* < 0.001
	-	1,144	8.90 ± 1.98		1,036	5.49 ± 1.69		1,036	−38.60	
**ApoB (g/L)**	+	42	1.89 ± 0.55	*p* = 0.166	19	1.13 ± 0.49	*p* = 0.755	10	−40.21	*p* = 0.122
	-	560	1.77 ± 0.54		343	1.09 ± 0.50		174	−37.75	
**TG (mmol/L)**	+	83	2.40 ± 1.53	*p* < 0.001	82	1.73 ± 0.95	*p* < 0.001	82	−28.04	*p* = 0.026
	-	1,134	1.74 ± 1.05		1,035	1.36 ± 0.76		1,026	−23.01	
**HDL-C (mmol/L)**	+	81	1.55 ± 0.41	*p* = 0.015	82	1.37 ± 0.41	*p* < 0.001	80	−11.20	*p* = 0.158
	-	1,129	1.67 ± 0.46		1,025	1.57 ± 0.46		1,012	−6.48	
**Lp(a) (g/L)**	+	65	0.60 ± 0.84	*p* = 0.059	23	0.88 ± 1.33	*p* = 0.042	23	5.32	*p* = 0.819
	-	887	0.45 ± 0.59		266	0.55 ± 0.66		261	4.53	

DM, diabetes mellitus; N—number of patients; SD, standard deviation; *p*—*p*-value.

In the DM+ subgroup (*n* = 83), 34 patients had a history of ASCVD (41%) whereas ASCVD was not present in 49 (59%). Among the DM–patients (*n* = 1,153), ASCVD was present in 122 (10.6%), with 1,031 patients (89.4%) without this diagnosis. The prevalence of ASCVD in DM+ vs. DM-was 41% vs 10,6%; *p* < 0.001 (see Table 7). In DM+ is 2.84 greater chance for KVO (OR = 2.84; CI0.95 = (1.67; 4.83)) than in DM-.

**TABLE 7:** DM+/DM-patients developing ASCVD.

	DM	ASCVD +	ASCVD -	Total
**Count**	+	34	49	83
**Row Percent (%)**		40.96	59.04	
**Count**	-	122	1,031	1,153
**Row Percent (%)**		10.58	89.42	

The baseline lipid profile in smokers (*n* = 389) differed significantly only in TG levels, which were higher (2.05 ± 1.37 mmol/L) compared with non-smokers (1.66 ± 0.93 mmol/L) and in HDL-C levels (1.60 ± 0.45 vs. 1.69 ± 0.46 mmol/L). During follow-up, TG levels in smokers remained higher (1.54 ± 0.99 vs. 1.3 ± 0.63 mmol/L), with the trend in HDL-C levels also unchanged (1.49 ± 0.47 vs. 1.59 ± 0.45 mmol/L). The follow-up levels of LDL-C and TC were lower in smokers (LDL 3.09 ± 1.47 vs. 3.3 ± 1.62 mmol/L, and TC 5.28 ± 1.65 vs. 5.51 ± 1.7 mmol/L, respectively). In smokers, their LDL-C levels declined by 3.44 mmol/L (3.13 mmol/L in non-smokers), with TC levels decreasing by 3.71 mmol/L (3.41 mmol/L in non-smokers).

Among smokers, 19.3% were classified as ASCVD+ and 80.7% as ASCVD–; the respective figures for non-smokers were 9.6 and 90.4%. In group of smokers is 1.87 greater chance for KVO (OR = 1.87; CI0.95 = (1.29; 2.71)) than in nonsmokers.

The whole group of our patients included 841 women and 395 men. Compared with men, women started follow-up with lower levels of TG (1.7 ± 1.05 mmol/L vs 1.95 ± 1.18 mmol/L; *p* < 0.001) and higher levels of HDL-C (1.77 ± 0.46 mmol/L vs 1.44 ± 0.34 mmol/L; *p* < 0.001). Over time, TG levels were higher in women, 1.35 ± 0.76 mmol/L (1.47 ± 0.83 mmol/L in men; *p* = 0.016) as were HDL-C levels, 1.67 ± 0.46 mmol/L (1.32 ± 0.36 mmol/L in men; *p* < 0.001). The follow-up TC levels were higher in women (5.57 ± 1.67 mmol/L) than in men (5.14 ± 1.68 mmol/L; *p* < 0.001). The changes between the baseline and follow-up levels of the other parameters assessed were non-significant.

Clinical presentation of FH was seen in a total of 145 (12%) patients, with xantelasma palpebrarum diagnosed in 57 cases (5%), arcus lipoides corneae in 47 patients (4%) and tendon xanthomas in 41 patients (3%).

## Discussion

The 1,236 patients with analyzed data attended a single Prague-based clinic with a history spanning more than 50 years. The period of data collection is not exactly defined as our project was a retrospective analysis with data of the first patients recorded as early as the 1960s when Šobra founded the Center of Preventive Cardiology (Center hereinafter) (Šobra, 1970). Over the decades, the Center was being attended by a large number of patients with familial hypercholesterolemia; however, the duration of their follow-up has varied substantially as, while some patients have been taken care of for decades, the follow-up period of other patients has not been longer than 2 years.

Needless to say, an ideal scenario would involve a patient referred to the Center by their general practitioner for assessment and subsequent follow-up. In practice, however, some patients presenting for follow-up do not have complete medical records, do not present for routine blood tests or are simply lost to follow-up. This explains the differences in the numbers of patients whose data were available for analysis. Last but not least, an additional reason may be the different, or inconsistent, approach of individual physicians.

Recent studies have suggested that the only causal factor of ASCVD is dyslipidemia or, more exactly, LDL-C (Borén et al., 2020). In fact, the diagnosis of FH *per se* puts all our 1,236 patients into the category of at least high cardiovascular risk (Visseren et al., 2021); nonetheless, while some of them do develop ASCVD, others do not. This was why our project focused also on the differences between the two major groups (ASCVD+ vs ASCVD–) of FH patients.

During follow-up, all patient subgroups showed a significant decrease in the levels of LDL-C, TC, ApoB and TG. While the reason for the decrease in HDL-C levels over time remains unclear, its follow-up levels (1.56 mmol/L) were within the optimal range (van der Steeg et al., 2008). Until the advent of PCSK9 inhibitors, Lp(a) was traditionally seen as an important player in the atherosclerotic process independent of the other risk factors (O'Donoghue et al., 2019) and not modifiable by drug therapy (Sun et al., 2018). The levels of Lp(a) did not change significantly in our analysis of follow-up data. There is no doubt this is due to the fact that PCSK9 inhibitors were unavailable in the Czech Republic until the summer of 2018; hence, they could not have affected the outcomes of patients on follow-up. Other reasons include the small number of patients with baseline and follow-up data available and, also, the inconsistent approach by physicians many of whom simply failed to focus their attention on a parameter refractory to drug therapy.

As noted above, not all patients with FH develop premature ASCVD. We did suspect that the lipid profile of ASCVD+ patients would be associated with increased risk, which was eventually the case. Patients with ASCVD had higher baseline LDL-C and TC levels and lower HDL-C levels than ASCVD–patients. The most striking differences were observed in the baseline levels of TG and Lp(a), which were again higher in the ASCVD+ subgroup thus corroborating, together with lower HDL-C levels, the importance of residual cardiovascular risk (Hoogeveen and Ballantyne, 2021). The tide turned during follow-up with ASCVD+ patients showing significantly lower levels of LDL-C, TC and ApoB whereas the differences in TG and Lp(a) levels were non-significant. The reasons for the more favorable lipid profile in ASCVD+ patients are multiple. First and foremost, these at-risk patients (category of very high cardiovascular risk according to the guidelines (Visseren et al., 2021)) receive more attention by health care providers. Also, their target levels are more ambitious and, last but not least, patients with a history of cardiovascular disease are more likely to adhere to their recommended therapy and tend to comply with their physicians’ advice (Jackevicius et al., 2002).

As in the ASCVD+ subgroup, patients assigned to the CHD subgroup had significantly higher baseline levels of LDL-C, TC, TG and Lp(a) a lower HDL-C levels compared with patients without a history of ASCVD. Except for TG and Lp(a), the follow-up levels in the CHD subgroup were lower (in analogy to ASCVD–vs ASCVD+). A similar trend was noted in the stroke (*n* = 31) and PAD subgroups (*n* = 32); however, the differences were non-significant due to the small number of patients on follow-up.

When comparing the subgroups with different therapeutic status (N/N, N/Y, Y/Y), it came as no surprise that the largest decrease in the levels of LDL-C, TC, ApoB and TG was seen in the subgroup with therapy not initiated prior to follow-up in the Center (N/Y). Nonetheless, a significant decrease in the above parameters was also seen in the (Y/Y) subgroup suggesting that patients benefit already from receiving therapy in a specialized center adopting the most recent therapeutic strategies combined with an effort to achieve target levels. Patients not currently on therapy and not treated at the time of starting outpatient follow-up showed minimal decreases in the investigated parameters. The most frequent reason for failure to initiate therapy in a specialized healthcare facility was statin intolerance. The number of patients not receiving therapy after the PCSK9 inhibitors had been approved for the Czech market is currently smaller (Altschmiedova et al., 2020); however, providing more details on this issue is outside the scope of this paper. Other reasons for not instituting therapy drug include the patients’ unwillingness and/or reluctance to initiate therapy even after they had been informed about all the risks associated with untreated significant dyslipidemia.

A total of 27% of our patients had a history of arterial hypertension, a condition with a global prevalence estimated at 20–24% in years 1975–2015 (Zhou et al., 2017). In the Czech Republic, according to Cífková et al., the prevalence of hypertension declined from 47.1% in 1985 to 41.5% in 2016/17 (Cífková et al., 2020a). Diabetes mellitus was present in 7% of our cohort. The prevalence of diabetes in the Czech Republic was according to the same author about 8% in men and 5% in women (Cífková et al., 2020b). These results clearly show that familial hypercholesterolemia is a genetic disease whose incidence cannot be linked to a lifestyle. Patients with FH are not in higher risk of development of diabetes and AH. The lipid profile of them with AH and diabetes is worse because of higher TG and lower HDL and we assume that this trend is associated with the lifestyle of individuals.

Smokers totaling 389, i.e., 31% of our whole group of patients, initiated follow-up with higher baseline TG levels and lower levels of HDL-C than non-smokers; this fact remained unaltered during follow-up and is presumably associated with the lifestyle of these patients. However, the follow-up levels of LDL-C and TC were more favorable in smokers. Smokers also tended to respond better to therapy and showed greater decreases in LDL-C, TC and ApoB levels compared with non-smokers, likely due to their higher cardiovascular risk and^,^ consequently, more ambitious LDL-C targets (Visseren et al., 2021).

Patients with FH and a history of arterial hypertension, diabetes or tobacco smoking, experienced more cardiovascular events than those without the above conditions.

At baseline and throughout follow-up, women had lower TG levels and higher HDL-C levels compared with men. These differences may be due to their more consistent adherence of women to a healthy lifestyle. We also assessed overall changes in the investigated parameters prior to and during follow-up; however, no sex-related statistical significance was demonstrated.

Clinical presentations of FH such as tendon xanthomas, arcus lipoides corneae or xantelasma palpebrarum are currently less frequent than in the past. In a first-ever monograph on FH published in 1970, Šobra reported a 30% incidence of arcus lipoides corneae, 23% incidence of xantelasma palpebrarum and 10% of patients with some form of xanthomatosis (Šobra, 1970). By contrast, in a paper published in 2014 and reporting on patients currently treated in the same center, arcus lipoides corneae, xanthelasma palpebrarum and xanthomatosis diagnosed were in 3, 6, and 5% of patients, respectively (Ceska et al., 2014). The development of these clinical signs is associated not only with cholesterol levels but, also, with the period of time the body is exposed to these levels. Patients with a well-defined treatment plan initiated in a timely manner do not develop these clinical presentations or, in the opposite case, these regress or disappear completely (Ceska et al., 2014; Civeira et al., 2016). If comparing the current therapeutic options with those available more than 50 years ago, it comes as no surprise that tendon xanthomas, arcus lipoides corneae or xantelasma palpebrarum become less frequent. We consider our assessment of the clinical signs in the present paper only an estimate since the figures cover all patients treated since the 1960s and the final number is no doubt confounded by the above regression due to intensive lipid-lowering therapy.

During the 50 + years of follow-up, there have been some deaths; however, the exact numbers are unavailable as some of the deaths may not have been recorded.

## Conclusion

The present analysis confirmed the well-known fact that, while LDL-C is a causal risk factor of ASCVD, every effort should be made to modulate all the known risk factors posing a residual risk, even after achieving target LDL-C levels. Therapeutic modification of Lp(a) by promising new agents still under development as well as by PCSK9 inhibitors already introduced into clinical practice may have the potential to further reduce cardiovascular risk in the near future. Results of this project have suggested that patients with the diagnosis of FH do benefit from receiving therapy in a specialized center which was confirmed by ScreenPro FH project (Ceska et al., 2019).

## Data Availability

The raw data supporting the conclusions of this article will be made available by the authors, without undue reservation.
